# Methods of 3D printing models of pituitary tumors

**DOI:** 10.1186/s41205-021-00118-4

**Published:** 2021-08-31

**Authors:** Daniel Gillett, Waiel Bashari, Russell Senanayake, Daniel Marsden, Olympia Koulouri, James MacFarlane, Merel van der Meulen, Andrew S. Powlson, Iosif A. Mendichovszky, Heok Cheow, Nick Bird, Angelos Kolias, Richard Mannion, Mark Gurnell

**Affiliations:** 1 Department of Nuclear Medicine, Cambridge University Hospitals NHS Foundation Trust, Cambridge Biomedical Campus, Hills Road, Cambridge, CB2 0QQ UK; 2 Cambridge Endocrine Molecular Imaging Group, University of Cambridge, Addenbrooke’s Hospital, Hills Road, Cambridge, Biomedical Campus, Hills Road, Cambridge, CB2 0QQ UK; 3 Clinical Engineering, Cambridge University Hospitals NHS Foundation Trust, Cambridge Biomedical Campus, Hills Road, Cambridge, CB2 0QQ UK; 4 Department of Radiology, University of Cambridge, Cambridge Biomedical Campus, Hills Road, Cambridge, CB2 0QQ UK; 5 Division of Neurosurgery, Department of Clinical Neurosciences, University of Cambridge & Addenbrooke’s Hospital, Cambridge, CB2 0QQ UK; 6 Metabolic Research Laboratories, Wellcome-MRC Institute of Metabolic Science, University of Cambridge, National Institute for Health Research, Cambridge Biomedical Research Centre, Addenbrooke’s Hospital, Hills Road, Cambridge, CB2 0QQ UK

**Keywords:** 3D printing, Pituitary, PET/CT, MRI, Cost analysis, Clinical utility

## Abstract

**Background:**

Pituitary adenomas can give rise to a variety of clinical disorders and surgery is often the primary treatment option. However, preoperative magnetic resonance imaging (MRI) does not always reliably identify the site of an adenoma. In this setting molecular (functional) imaging (e.g. ^11^C-methionine PET/CT) may help with tumor localisation, although interpretation of these 2D images can be challenging. 3D printing of anatomicalal models for other indications has been shown to aid surgical planning and improve patient understanding of the planned procedure. Here, we explore the potential utility of four types of 3D printing using PET/CT and co-registered MRI for visualising pituitary adenomas.

**Methods:**

A 3D patient-specific model based on a challenging clinical case was created by segmenting the pituitary gland, pituitary adenoma, carotid arteries and bone using contemporary PET/CT and MR images. The 3D anatomical models were printed using VP, MEX, MJ and PBF 3D printing methods. Different anatomicalal structures were printed in color with the exception of the PBF anatomical model where a single color was used. The anatomical models were compared against the computer model to assess printing accuracy. Three groups of clinicians (endocrinologists, neurosurgeons and ENT surgeons) assessed the anatomical models for their potential clinical utility.

**Results:**

All of the printing techniques produced anatomical models which were spatially accurate, with the commercial printing techniques (MJ and PBF) and the consumer printing techniques (VP and MEX) demonstrating comparable findings (all techniques had mean spatial differences from the computer model of < 0.6 mm). The MJ, VP and MEX printing techniques yielded multicolored anatomical models, which the clinicians unanimously agreed would be preferable to use when talking to a patient; in contrast, 50%, 40% and 0% of endocrinologists, neurosurgeons and ENT surgeons respectively would consider using the PBF model.

**Conclusion:**

3D anatomical models of pituitary tumors were successfully created from PET/CT and MRI using four different 3D printing techniques. However, the expert reviewers unanimously preferred the multicolor prints. Importantly, the consumer printers performed comparably to the commercial MJ printing technique, opening the possibility that these methods can be adopted into routine clinical practice with only a modest investment.

## Background

The pituitary gland plays a critical role in regulating the body’s response to a variety of internal and external stimuli. This is achieved through secretion of several key hormones including adrenocorticotropic hormone (ACTH), thyroid stimulating hormone (TSH), growth hormone (GH), luteinizing hormone (LH) and follicle-stimulating hormone (FSH). These hormones are released into the circulation and, in turn, direct hormone release from key target tissues (e.g. thyroid, adrenal, ovaries/testes, liver), thus modulating an array of physiological processes, including cardiovascular function, metabolism and reproductive function. Pituitary hormone release is subject to negative feedback in which target hormones, acting at both pituitary and hypothalamic levels, diminish central hormone release. Autonomous (unregulated) hormone production from a functioning pituitary adenoma (PA, non-cancerous pituitary tumor) can perturb this delicate equilibrium, manifesting a variety of clinical syndromes including ACTH-dependent Cushing’s syndrome (excess ACTH), acromegaly (excess GH), hyperprolactinaemia (excess prolactin), and hyperthyroidism (excess TSH). The associated clinical symptoms can have a devastating impact on a patient’s quality of life and some of these syndromes are also associated with higher mortality rates [1].

Surgery remains the mainstay of treatment for several subtypes of PA. This is typically undertaken via the transsphenoidal route, which offers both a safe and effective approach [2, 3]. However, incomplete resection can occur depending on various factors, including adenoma size [4, 5] and proximity to adjacent vital structures such as the carotid arteries contained within the cavernous sinuses, which are located on either side of the pituitary gland [6, 7]. Additionally, transsphenoidal surgery (TSS) is not without risk, with complications including cerebrospinal fluid (CSF) leak and visual deterioration in the acute phase, and hypopituitarism (ie loss of pituitary gland function) in the longer term [8]. Safe and effective surgery is therefore heavily reliant on high quality preoperative imaging to allow accurate localisation of the adenoma. Magnetic resonance imaging (MRI) is the preferred modality to assess the pituitary gland and surrounding structures [9]. However, there are circumstances when the utility of MRI in preoperative assessment is limited, in particular adenomas less than 10 mm (microadenomas) and/or following recent pituitary surgery, where it can be difficult to distinguish normal pituitary gland from residual adenomatous tissue and post-surgical changes [10]. Alternative strategies to aid visualization of the pituitary gland have been developed, in particular hybrid Positron Emission Tomography (PET) with x-ray Computed Tomography (PET/CT) [11–13] or MRI (PET/MR) [14, 15] which permit correlation of function and anatomy. We have previously described the utility of [^11^C]-methionine in Cushing’s syndrome [11], acromegaly [16] and TSHoma [17].

Before deciding a patient’s management plan, pituitary imaging is typically reviewed by a multidisciplinary team (MDT), comprising of clinicians from various disciplines, including endocrinology, neurosurgery and otolaryngology [ear, nose and throat (ENT)], to determine suitability for surgery and inform the choice of surgical approach [18]. In addition, these images are also used during discussion with the patient preoperatively to explain the proposed treatment. However, such 3D visualizations of the pituitary gland and associated pathology may give rise to several challenges: from the surgical perspective, it can be difficult to fully appreciate adenoma location and proximity to adjacent critical structures, even more so in surgical revision cases; from a patient perspective, it is often difficult to relate the 3D visualizations to their own anatomy.

3D printing, based on cross-sectional imaging findings, have begun to find use in other surgical disciplines to inform surgical planning and enhance patient understanding [19]. Importantly, guidelines have been developed for the use of 3D printing in clinical practice, including for cardiac, hepatobiliary, gastrointestinal and other conditions where it may have a potential role in informing management [20–22]. However to our knowledge these guidelines do not make specific mention to the pituitary gland although a small number of studies have shown the feasibility of 3D printing of pituitary tumors [23–25].

Here, we build on this work and demonstrate how findings from anatomical and molecular (functional) imaging can be combined to produce 3D printed patient-specific models. Based on a single complex pituitary tumor case, we compare 3D printed models from both commercial and consumer printers with a view to enabling early, cost-effective adoption into routine clinical practice.

## Methods

### Imaging

MRI was performed on a GE Optima™ MR450w 1.5 T scanner (GE Healthcare, Chicago, Illinois, United States) using a Fast Spoiled Gradient Echo (FSPGR) sequence to create a 3D volumetric dataset with 1 mm^3^ voxels and 1 mm spacing. PET/CT was performed using a GE Discovery 690 scanner (GE Healthcare, Chicago, Illinois, United States). The PET scan was acquired for 20 min at 20 min post administration of 390 MBq of [11C]-methionine (Wolfson Brain Imaging Centre, Cambridge). The images were reconstructed using GE’s Sharp Iterative Reconstruction (SharpIR) algorithm with CT measured attenuation correction, scatter correction and time of flight using 2 iterations and 24 subsets with a 3.2 mm Gaussian filter. The CT scan acquired with 140 kV, fixed mA of 220, a rotation speed of 0.5 s, a pitch of 0984:1, 30 cm field of view, a slice thickness of 1.25 mm and a 1.25 mm spacing interval. The CT was reconstructed using filtered back projection.

### Image segmentation

To prepare the images for segmentation, the CT was registered to the volumetric MRI using 3D Slicer [26] (version 4.10.2, 05–2019). A rigid registration was used with 6 degrees of freedom, maximum number of iterations of 1500 and a sampling ratio of 0.1%. The resulting transformation was applied to the PET images so that all three imaging modalities were co-registered (PET/CT + MR).

**Fig. 1:**
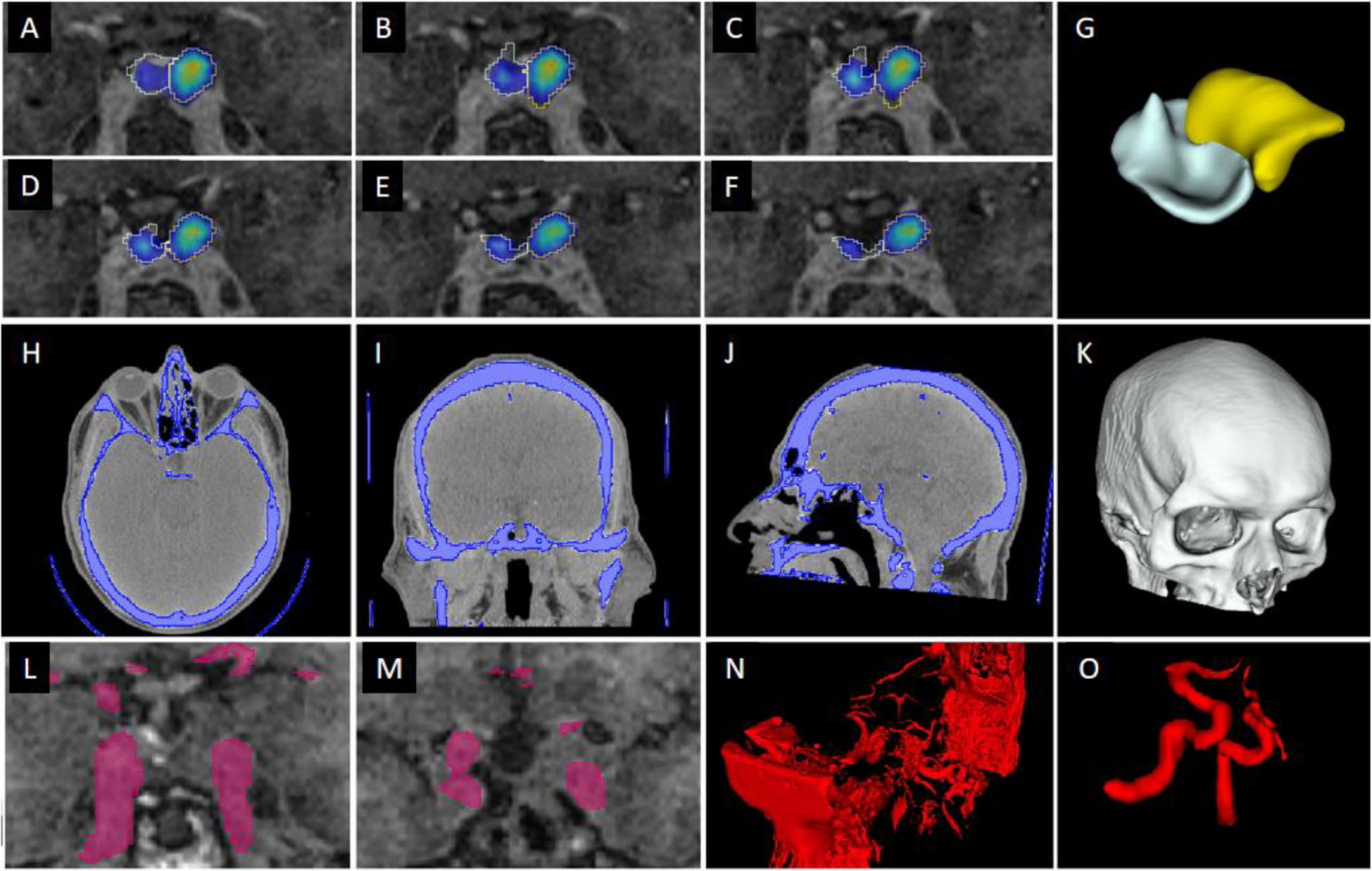
Segmentation of the pituitary gland and adjacent structures. **A–F,** Pituitary gland (blue) and pituitary tumor (yellow) segmentation using PET registered with MRI. **G,** 3D rendered model of pituitary gland (white structure) and pituitary tumor (yellow structure). **H–J,** Bone segmentation from CT using thresholding tool. **K,** 3D rendered model of bone after removal of small islands and imaging bed. **L–M,** Carotid arteries segmentation with FSPGR MR using thresholding tool. **N,** Initial 3D rendered segmentation of MR soft tissue. **O,** Final segmentation of carotid arteries after unwanted structures have been removed

Using 3D Slicer the pituitary gland and tumor were segmented on the MRI with the PET image overlaid (Fig. 1). This segmentation was a predominantly manual process that was guided by the PET and with pituitary regions-of-interest drawn on to each slice of the MRI.

The carotids were outlined using a voxel thresholding tool on the MRI. The initial carotid segmentation included some unwanted structures which were removed using the cutting tool in 3D Slicer. Only vessels adjacent to the pituitary gland and tumor (within approximately 30 mm) were included in the model (Fig. 1).

The skull was segmented from the CT using voxel Hounsfield unit (HU) values between 100 HU and the maximum HU in the image (Fig. 1). Small floating structures within this segmentation were removed using the ‘Islands’ tool and the imaging bed was also removed.

The final segmentations were agreed together with expert reviewers with experience in neuroradiology and molecular imaging, specifically [^11^C]-methionine PET interpretation, to ensure that the boundaries of the segments conformed to the corresponding anatomicalal structures (using MR and CT) and functional uptake on PET.

### Model preparation for 3D printing

All the structures were smoothed using a median smoothing filter of 3 mm. The model was limited to the structures between the centre of each orbit, inferior to a horizontal plane superior to the supraorbital ridge. The part of the skull base posterior to the clivus was also removed. This process limited the amount of material required for the print but retained all of the structures needed for surgical planning such as the nasal cavity and the bones at the base of the skull surrounding the pituitary gland.

**Fig. 2:**
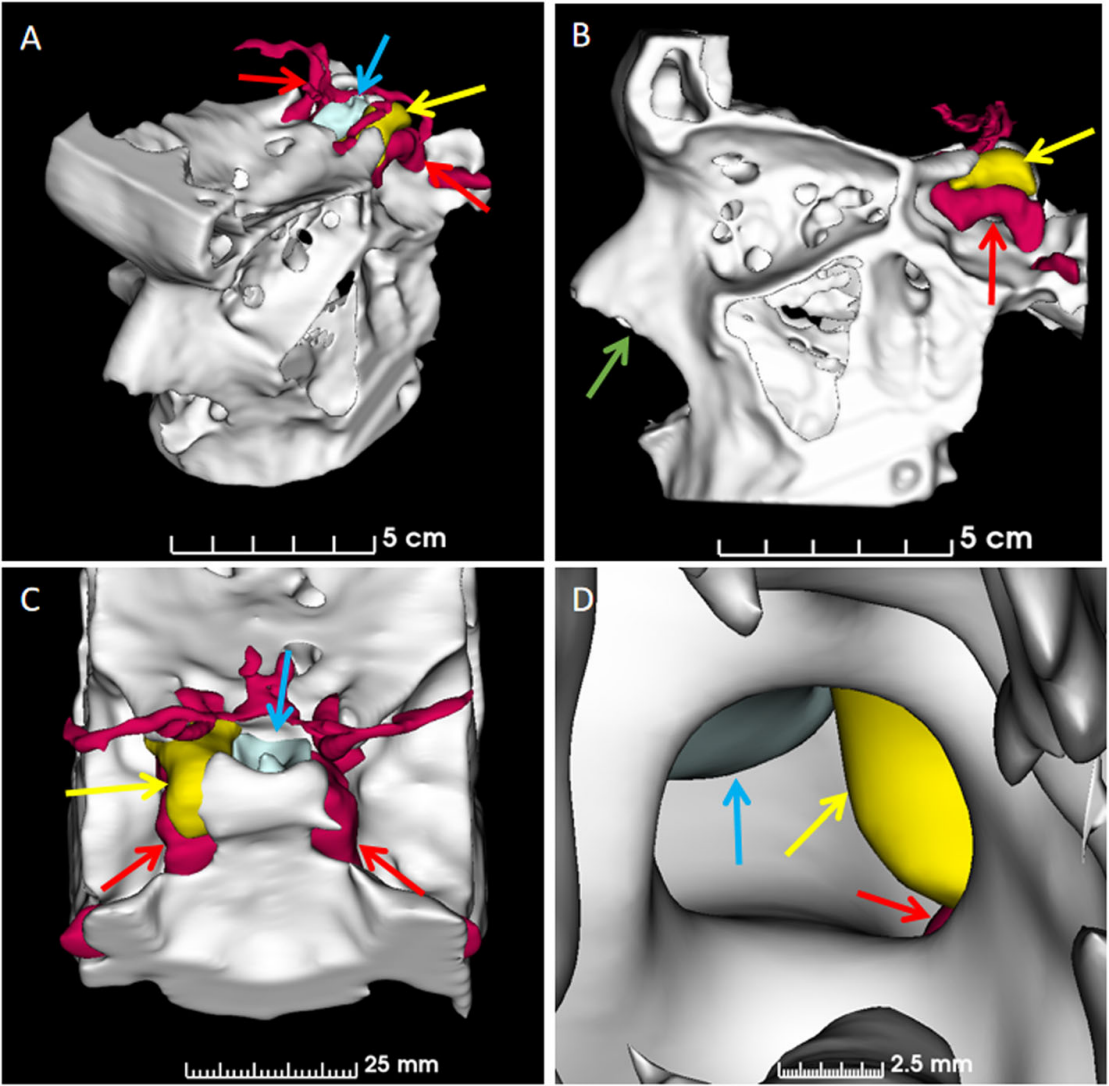
Final 3D rendered model. **A,** Oblique view, **B,** lateral view, **C,** postero-superior view and **D,** view through the previous surgical opening (which has left a bony defect) in the floor of the pituitary fossa. Blue arrows indicate location of normal pituitary gland, yellow arrows indicate location of adenoma and red arrows indicate location of carotid arteries. The green arrow indicates the trajectory of the surgical approach via the trans-nasal route

The resulting individual segmentations were brought together using 3D Slicer to form a final model comprising the pituitary gland, pituitary tumor, carotids and surrounding bone (Fig. 2). The final model was prepared differently depending on the type of printing technique. For powder bed fusion (PBF) printing, the segments were added together and printed monochromatically (white). Multi-material material extrusion (MEX) and material jetting (MJ) are multicolor techniques and for these each segment was assigned a color during the printing setup (pituitary gland [blue], adenoma [yellow], carotids [red], bone [white]). Monocolor vat photopolymerization (VP) permits the use of transparent resin; accordingly, the pituitary gland, pituitary tumor and carotid segments could be made hollow to allow introduction of colored resin after printing. The hollowing process was automated by specifying the wall thickness (0.5 mm) and position of the wall (median surface). This process resulting in a 0.25 mm internal and a 0.25 mm external thickening of the boundary. After combining the segments small discrete holes were created in the pituitary gland, tumor and carotid segments to facilitate the addition of the different colored resin. The final versions of the model for all printing techniques were exported from 3D Slicer as Standard Tessellation Language (STL) files.

### 3D printing techniques

The PBF anatomical model was printed on a EOS100 printer with a resolution of 0.1 mm using Nylon PA2200 by an external commercial provider (3DPRINTUK, Leyton, UK). The STL file was uploaded to the website and it was subsequently printed using a single color (white) nylon material.

The MJ anatomical model was printed on a Stratasys J750 printer with a layer height of 0.014 mm by an external supplier (Laserlines, Banbury, UK). The STL files were sent to the company with each structure being assigned a different color (bone - white, pituitary - blue, pituitary tumor - yellow, carotids - red). These same colors were also used for the MEX and VP anatomical models.

**Fig. 3:**
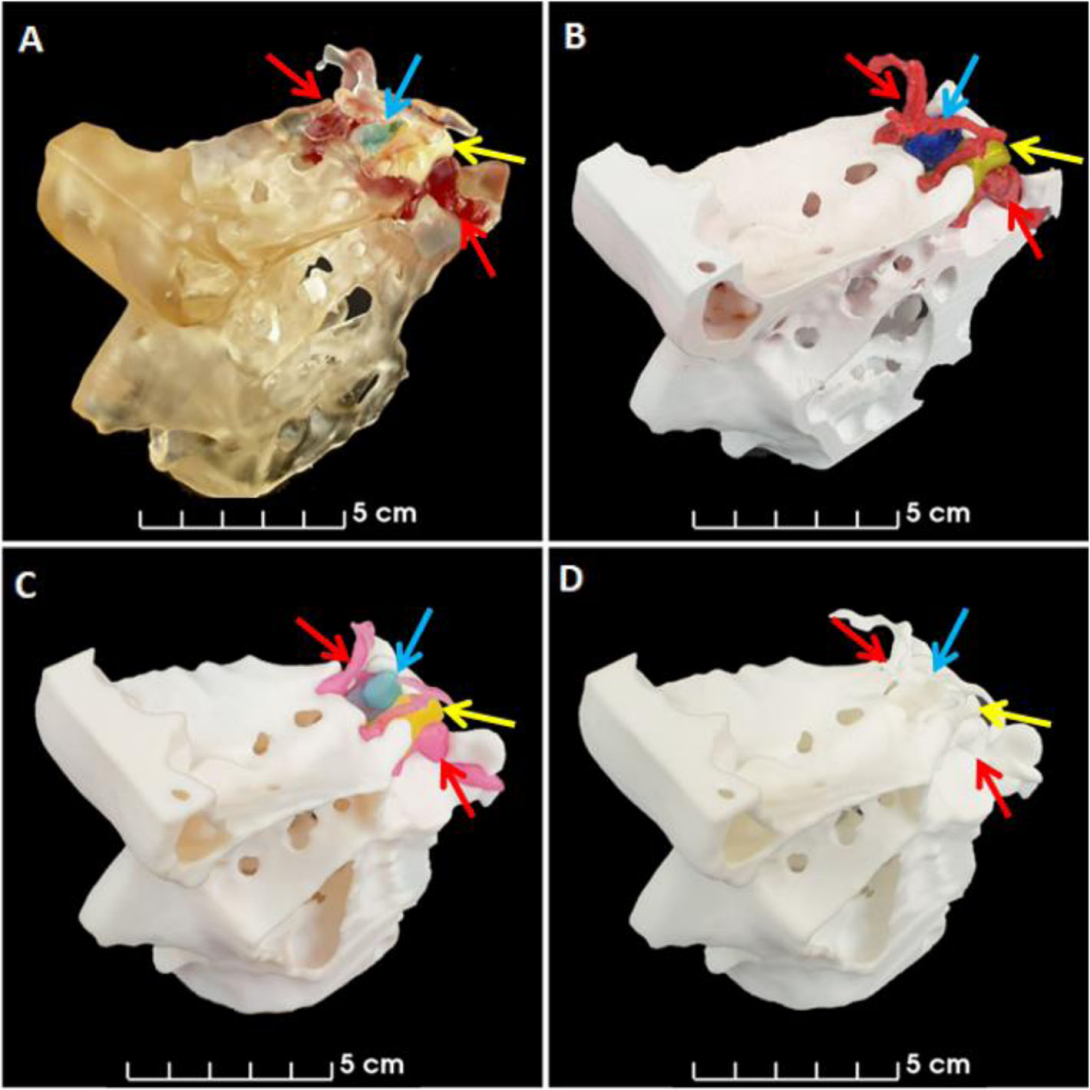
Angled views of anatomical models. Images of **A,** VP, **B,** MEX, **C,** MJ and **D,** PBF anatomical models. Blue arrows indicate location of pituitary gland, yellow arrows indicate location of adenoma and red arrows indicate location of carotid arteries

**Fig. 4:**
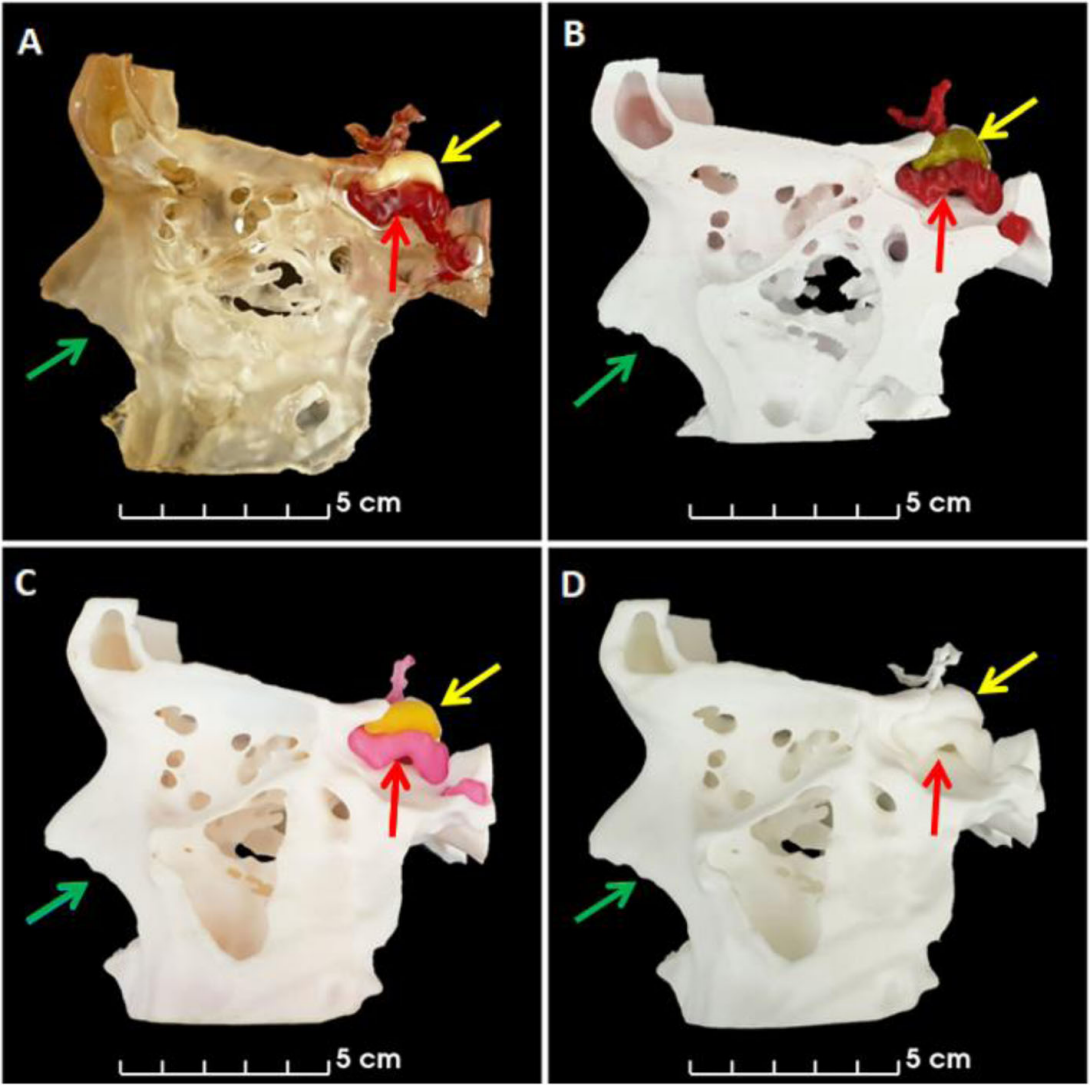
Lateral views of anatomical models. Left lateral images **A,** VP, **B,** MEX, **C,** MJ and **D,** PBF anatomical models. Yellow arrows indicate location of adenoma and red arrows indicate location of carotid arteries. Green arrows indicate where the surgeons enter the nose to access the pituitary gland through the pituitary sella, which is shown in Fig. 6 for all anatomical models

**Fig. 5:**
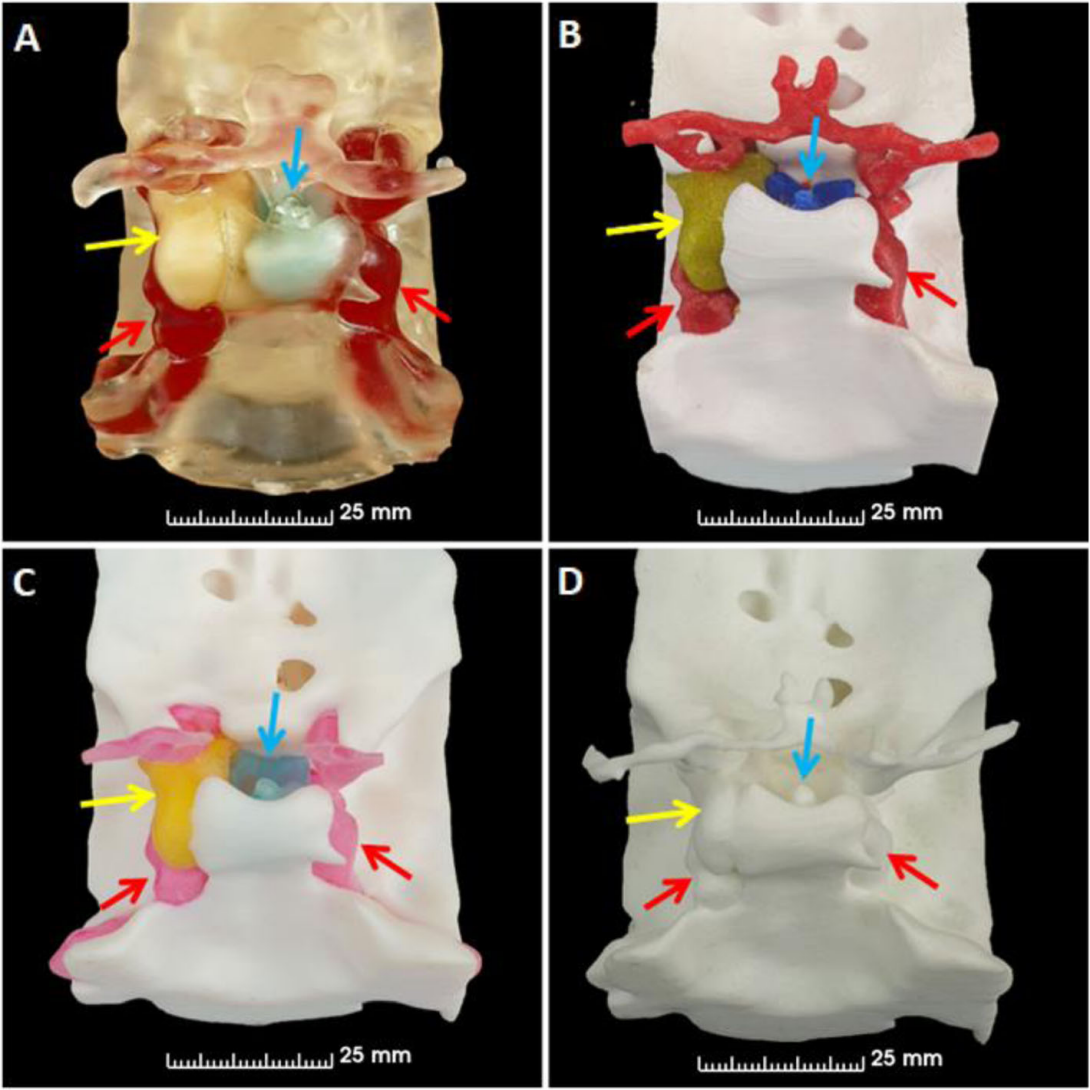
Posterior views of anatomical models. Posterior images of **A,** VP, **B,** MEX, **C,** MJ and **D,** PBF anatomical models. The blue arrows indicate location of pituitary gland, the yellow arrows indicate location of adenoma and the red arrows indicate location of carotid arteries

**Fig. 6:**
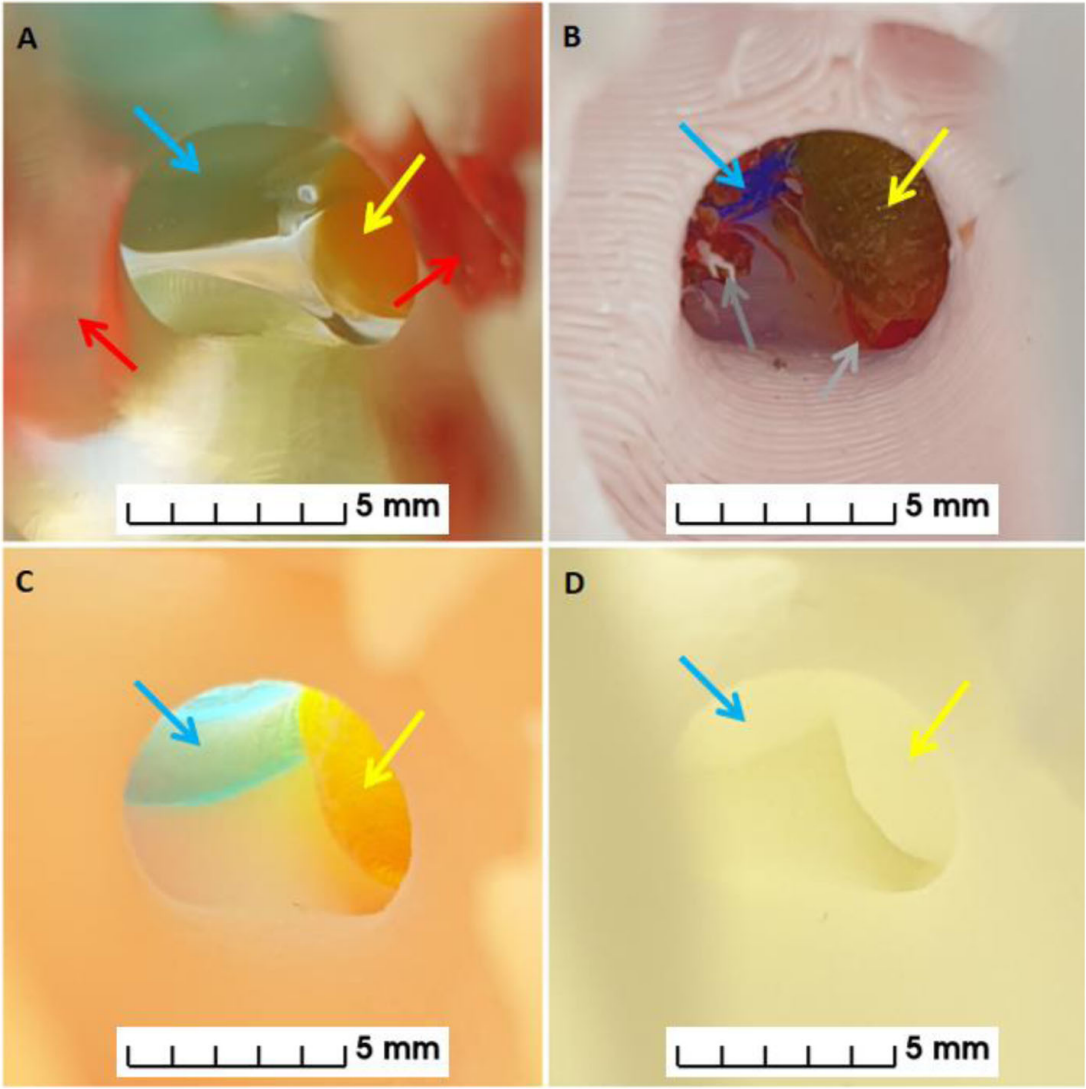
Views from the nose (as per the transsphenoidal surgical approach) of the anatomical models. Images from the nose of **A,** VP, **B,** MEX, **C,** MJ and **D,** PBF anatomical models. The blue arrows indicate the location of the pituitary gland and the yellow arrows indicate the location of the adenoma. The red arrows on panel **A** highlight that the carotid arteries are visible through the transparent bone of the VP anatomical model. The grey arrows on panel **B** highlight that the remnant support structures are still visible through opening in bone on the MEX anatomical model

MEX printing was carried out at our institution using a Prusa Research MK3 with a Multiple Material Unit (Prusa Research, Prague, Czech Republic). The STL files were prepared for printing using PrusaSlicer and exported as gcode. Four colored PETG filaments were used to print using a layer height of 0.2 mm and a 0.4 mm nozzle resulting in a print time of 24 h (Figs. 3, 4, 5 and 6). PETG was preferred over PLA to minimise the risk of color fading with time. The anatomical model was printed with support structures which were removed afterwards.

VP printing was carried out in-house using a Prusa Research SL1 (Prusa Research, Prague, Czech Republic) and a transparent resin. The STL files were prepared for printing using PrusaSlicer and exported as masked SLA files (.SL1 files). A layer height of 0.05 mm with exposure times of 35 s and 6 s were used for the initial layers and subsequent layers respectively. Printing took eight hours. Immediately after the printing finished the anatomical model was washed in Isopropyl Alcohol 99% (IPA) for 10 min. The anatomical model and its support structures were then removed from the printing plate, dried using hot air for five minutes and cured with ultraviolet radiation (UV) for five minutes. The voids were then filled with colored printing resin using a syringe and a 19 gauge needle before being cured again for another five minutes (Panel A in Figs. 3, 4, 5 and 6). The washing, drying and curing were all carried out using a Prusa Research CW1 (Prusa Research, Prague, Czech Republic).

### Anatomical model evaluation

Each anatomical model was evaluated for i) accuracy and ii) perceived clinical utility.

**Fig. 7:**
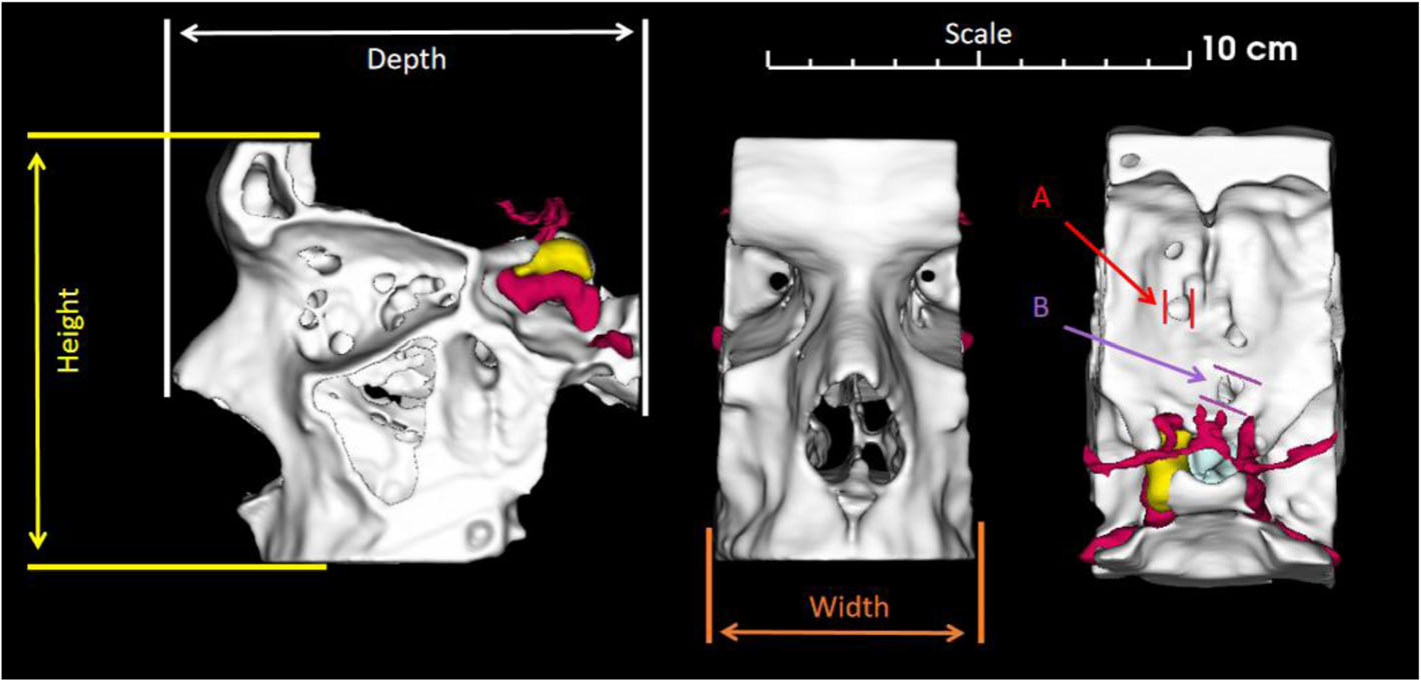
Assessment of printing accuracy. The five distances used to assess printing accuracy were the depth, height and width of the models, together with two small (< 1 cm) landmarks in the skull base (A and B). The depth, height and width were determined as shown with a height gauge. Both small landmarks were measured using callipers

i) Accuracy was assessed through comparison of five measurements (height, width and depth of the models and diameters of two small landmarks in the skull base) determined on the anatomical models and on the computer model (using 3D slicer) see Fig. 7 for measurement locations. Assessment of the printed anatomical models was performed by two blinded operators (DG and DM) using a calibrated height gauge and calibrated calipers. The operators came to a consensus on each measurement. The differences between the computer model and the printed anatomical models were used to assess printing accuracy.
ii) Clinical utility was assessed by three groups of clinicians (endocrinologists, ENT surgeons and neurosurgeons) by filling out a questionnaire. The following questions and answer scaling were used:

1. How useful do you think this model would be in informing the patient about their disease? (Very Poor [1], Poor [2], Acceptable [3], Good [4], Very Good [5])
2. How useful do you think this model would be in informing the patient about their surgery? (Very Poor [1], Poor [2], Acceptable [3], Good [4], Very Good [5])
3. Do you think the use of a model like this would change patient care? (Definitely not [1], Probably not [2], Possibly [3], Probably [4], Definitely [5])
4. How useful do you think this model would be for training other clinicians/surgeons? (Very Poor [1], Poor [2], Acceptable [3], Good [4], Very Good [5])
5. Overall how would you rate the quality of the model? (Very Poor [1], Poor [2], Acceptable [3], Good [4], Very Good [5])
6. Would you like to use a model like this when talking to a patient? [Yes or No]
7. How much would you pay for this model? (£)
8. Any additional comments

The results for questions 1 to 6 were visualised and compared using the Likert function in the statistical package R [27]. For Question 7, the average amount that the clinicians would be prepared to pay was compared for each model.

## Results

### Model preparation

Segmenting the structures of interest (Fig. 1) took approximately 90 min, with the majority of this time being spent on manually segmenting the pituitary gland and the pituitary adenomas (see Fig. 1 panels A-G). In contrast, the bone segmentation was performed using a semi-automated threshold technique with the only manual intervention being to define the extent of the surrounding bone included in the final print (see Fig. 1 panels H-K). The process was similar for the carotid arteries but more manual intervention was required because of the limited contrast between the arteries and the surrounding tissue on MRI (see Fig. 1 panels L-O).

For the VP anatomical model, additional steps were required to allow the creation of hollow structures. A shell thickness of 0.5 mm was centred on the surface of the structure. This resulted in the boundary moving outwards 0.25 mm and inwards 0.25 mm which provided the optimal compromise between the size of the cavity for resin injection versus encroachment on adjacent structures.

### 3D printing

The commercial printing options - MJ and PBF - were simple to use as they did not require any knowledge of the printing processes which were outsourced. In contrast, the consumer printing options - VP and MEX - required local expertise/knowledge and as a result the consumer printers were associated with additional preparation costs and required trained staff.

The consumer printers - VP and MEX (Figs. 3A-B,4A-B,5A-B and 6A-B) - were able to produce anatomical models more quickly than the commercial printers - MJ and PBF (Figs. 3C-D,4C-D,5C-D and 6C-D) - because they could be used on site whereas the commercially printed models had to be posted to us. The VP, MEX, MJ and PBF anatomical models were produced and received in 0.3, 1.0, 30 and 14 days, respectively.

### Cost

The cost per model for the consumer printers - VP and MEX - was calculated by factoring in the time required by technical staff to set up the printer and finish the print, the cost of materials and the cost of the printer itself.

For the VP and MEX anatomical models, part of the estimated costs was based on each method taking approximately two hours of a technician’s time. For MEX printing this time is split between setting up the printer for the desired materials and starting the printing (30 min) and removing the support structures (90 min). However, if dissolvable support structures could be used, this time, and therefore cost, could be reduced. For the VP printing this time was divided between setting up the printer with the material (15 min), washing and curing the printed anatomical model (30 min), removing the support structures (30 min) and filling the hollow structures (45 min).

The mean cost per model when the cost of the printer itself is taken into account is therefore affected by the number of models each printer produces. As this is an unknown quantity, 10 and 100 prints have been used to allow for an arbitrary comparison, representing a relatively low and relatively high usage. The cost per model for the commercial printing techniques - MJ and PBF - was simply taken as the price paid for the models. As a result the VP and MEX techniques have potential ranges of £76 to £220 and £70 to £160 respectively. The PBF technique is comparable in cost to consumer printers whereas the MJ printer is approximately double the cost. The data is summarised in Table 1.

**Table 1:** Cost analysis. Cost for printing preparation and finishing assumed to be £25 per hour. The materials differ between the VP and MEX but the costs are approximately the same per anatomical model. The estimated cost per model for the low-cost consumer printers - VP and MEX - is based on each printer printing 10 or 100 models

Cost analysis (£)	VP		MEX		MJ	PBF
Printing prep and finishing	50.00		50.00		*N/A*	*N/A*
Materials	10.00		10.00		*N/A*	*N/A*
Price paid for anatomical model	*N/A*		*N/A*		534.00	120.00
Printer cost	1600.00 *(Prusa, SL1)*		1000.00 *(Prusa, Mk3, MMU2)*		*N/A*	*N/A*
Estimated cost per print	76.00 (100 models)	220.00 (10 models)	70.00 (100 models)	160.00 (10 models)	534.00	120.00

Key: *MEX* Material Extrusion, *MJ* Material Jetting, *PBF* Powder bed fusion, *VP* Vat Photopolymerization.

### Print accuracy

**Fig. 8:**
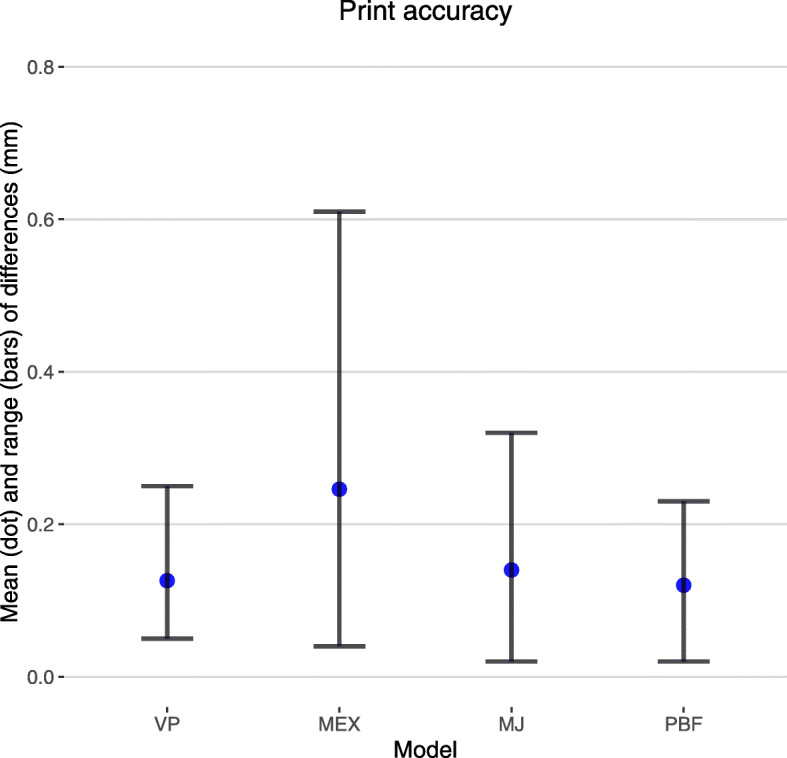
Print accuracy. The differences in measurements (mm) between computer models and printed anatomical models are shown as means (blue dots) and ranges (black bars) for each printing technique

The accuracy of the printing dimensions was assessed in five positions (see Fig. 7). Figure 8 shows the mean and range of the differences taken from the printed anatomical models and the computer models (a single one anatomical model used for each printing technique). The maximum differences were less than 0.7 mm for all models.

The PBF anatomical model had excellent printing accuracy and was the only anatomical model that had no visible layer lines and no imperfections. The MJ and VP anatomical models had almost as good printing accuracy and only had visible layer lines when the anatomical models were inspected closely (see Fig. 6 - panel B). However, the VP anatomical model had visible imperfections where the resin was added to the hollow cavities and on the MJ anatomical model the anterior cerebral arteries were easily damaged as they were very slender. The MEX anatomical model had easily visible layer lines and the worst spatial accuracy but it was still well within the predefined 1 mm accuracy threshold (see Fig. 7). Unfortunately this anatomical model had visible remnants of the internal support structures where they were not able to be completely removed, as indicated in Fig. 6 panel B.

### Perceived clinical utility

The PBF anatomical model had the lowest rating for questions 1–6 from all three groups of clinicians. The PBF anatomical model was the only monocolor anatomical model and this was given as the reason for its poor performance in the questionnaire comments.

On average the multicolor anatomical models were all rated positively for usefulness in informing the patient about their disease. The MEX anatomical model was favoured by the endocrinologists and the VP was favoured by the neurosurgeons and ENT surgeons.

As a general rule, the multicolor anatomical models were rated positively for their usefulness in informing the patient about their surgery. The MJ anatomical model received the highest score from the Endocrinologists and the VP anatomical model was awarded the highest score by the neurosurgeons and ENT surgeons.

The endocrinologists and ENT surgeons concluded that all three multicolored anatomical models could potentially change patient management. However, the neurosurgeons adopted a more neutral position, indicating it was possible but not probable they would change patient management; they did not express a preference with all three multicolor anatomical models scoring the same.

The multicolor anatomical models were rated positively for their potential utility in training other clinicians. The MEX and MJ anatomical models both had the highest rating from the endocrinologists, whereas the VP anatomical model was preferred by both the ENT surgeons and the neurosurgeons.

From a print quality perspective, the colored anatomical models were preferred, with the MJ anatomical model rated highest by the Endocrinologists and the VP anatomical model rated highest by the ENT surgeons and neurosurgeons.

All clinicians indicated they would welcome the opportunity to have any of the multicolored anatomical models available when consulting with a patient.

**Fig. 9:**
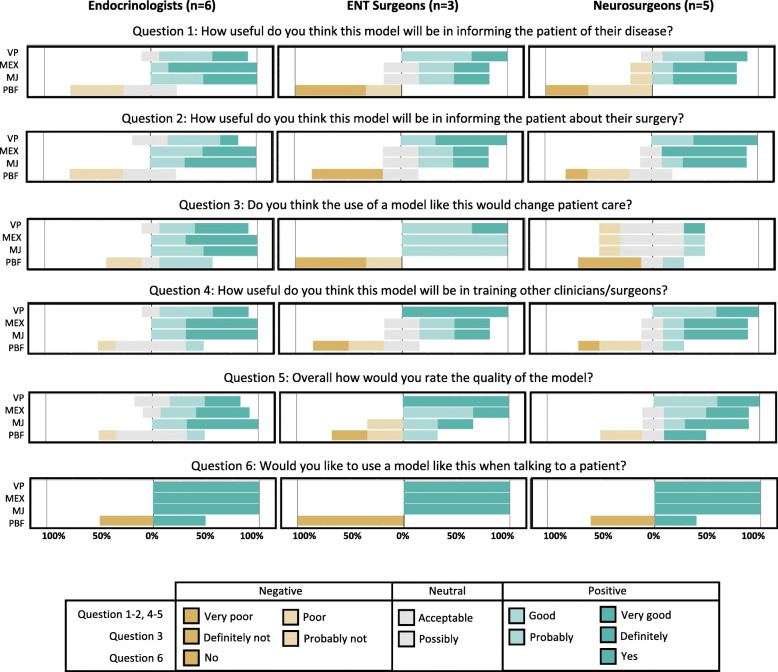
Responses to questionnaire. The position of each horizontal bar indicates the relative weighting of negative (1–2), neutral (3) and positive (4–5) responses, with the bar moving progressively further to the right as the number of positive responses increased. The colors of the bars denote the proportion of the responses which were assigned to each option on the respective Likert scales as shown in the panel at the bottom of the figure

The results of questions 1–6 of the questionnaire are summarised for the endocrinologists (*n* = 6), ENT surgeons (*n* = 3) and neurosurgeons (*n* = 5) in Fig. 9.

The results of question 7 (estimating the monetary worth of each anatomical model) were combined for all expert groups and are shown in Table 2.

**Table 2:** Mean estimates of monetary worth for each anatomical model

Results of question 7 (£)	VP	MEX	MJ	PBF
Mean worth and range according to clinicians	82 (10 to 400)	83 (15 to 300)	88 (20 to 300)	45 (0 to 200)

## Discussion

We have shown that consumer printers are able to produce accurate 3D printed anatomical models of pituitary tumors segmented using a combination of structural (CT and MRI) and functional ([^11^C]-methionine PET) imaging.

In a previous study of patients with malignant brain tumors (glioma), a 3D printing approach was shown to improve patient understanding and support clinical decision making [28]. For patients with pituitary tumors 3D printed anatomical models have been proposed for enhancing surgical training [23, 24]. A particular focus of these early models has been the visual appearance and aesthetics with respect to mimicking human tissue. Other workers have explored the use of 3D printed patient-specific models to inform surgical planning, and have demonstrated benefits in terms of operative duration and volume of blood loss in a group of 10 patients (compared with control subjects undergoing surgery not informed by a 3D anatomical model) [25].

We have previously reported the potential utility of 3D rendered models, which combine data from anatomicalal imaging (MRI and CT) with molecular (functional) imaging using [^11^C]-methionine PET/CT, in the management of patients with pituitary tumors [29]. Specifically, in patients with persistent disease following initial surgery, and in whom there is uncertainty regarding the resectability of residual tumor, we have shown that 3D rendered models can aid distinction between the tumor and surrounding normal tissues and inform the surgical approach [26].

Segmenting the structures of interest involved manually drawing around the pituitary gland and tumor (Fig. 1 panels A-F). This process was aided by incorporation of PET datasets, co-registered to the MRI, thus allowing the active tumor (often best seen on the functional imaging) to be more readily distinguished from postoperative scar tissue. Semi-automatic segmentation tools, such as thresholding, could not differentiate between the pituitary gland, pituitary adenomas and surrounding tissues. However, the use of artificial intelligence-driven segmentation, which has shown promise in brain tumors including pituitary tumors [30], may allow this process to be automated. In contrast, bone was readily segmented (See Fig. 1 panels H-K) and we anticipate this aspect could be completely automated in future studies. The bone was a key part of the final model, which emphasises the need for CT - or ultrashort echo time MR sequences - to permit bone segmentation. At the time of the study, the commercial printing techniques - MJ and PBF - had lead times of 2 weeks or more and, accordingly, if they were to be adopted into routine clinical practice this delay would need to be taken into account. However, many printing services now offer lead times of 2–4 days, which would minimise any potential impact on clinical decision-making. These printing techniques also have advantages over the consumer printing techniques - VP and MEX - because they are readily accessible and do not require the institution to have specialist equipment or staff trained in 3D printing. In contrast, the consumer printing techniques offer the advantage of more rapid turnaround (less than 2 days) of the appropriate imaging being obtained. Another important consideration in assessing the feasibility of these printing techniques was the accuracy of the printed anatomical model compared to the computer model. The measurements taken from the anatomical models (see Fig. 7) demonstrated that all of the techniques had mean differences of less than 0.5 mm (see Fig. 8) with the PBF, VP and MJ anatomical models having marginally smaller differences to the MEX anatomical model.

The VP technique required additional manipulations to deliver a multicolored anatomical model, which involved two distinct steps: firstly, segmentations of the carotid arteries, normal pituitary gland and tumor were hollowed out and added to the bone segment to create one object that could be printed. This allowed the resulting structure to be printed without internal supports and to be filled with liquid resin. To ensure the hollow structures were printed successfully, it was necessary to strike a balance: the shell needed to be thick enough to support itself during printing but not too thick (otherwise the internal cavity would be too small). In addition, a small discrete hole was also required to enable injection of the colored resin. After each cavity was filled the model required curing with UV radiation to solidify the colored resin. Importantly, the model was cured in an orientation that would not allow the resin to flow out of the cavity. These two steps therefore added additional complexity and time to the creation of this model.

The MJ and MEX anatomical models had a similar appearance with both incorporating solid colors depicting the tissues of interest (see Figs. 3, 4, 5 and 6 panels B and C). However, remnants of the support structures detracted from the overall appearance of the MEX anatomical model (Fig. 6 panel B). To overcome this problem, future models could be printed with dissolvable supports [31]. However, this would require (i) the use of support material that is compatible with the thermoplastic, such as PolyVinyl Alcohol (PVA) and (ii) a MEX printer capable of using multiple materials. The VP anatomical model used similar colors but had a different appearance because the bone was transparent and the pituitary gland, tumor and carotid arteries were all slightly enlarged by the process of hollowing them out (see Figs. 3, 4, 5 and 6 panel A). Lastly, the PBF anatomical model lacked color and consequently contrast between the tissues of interest (see Figs. 3, 4, 5 and 6 panel D). Whilst the anatomical model could be painted after printing, this process would be dependent on the interpretation of the painter which could result in errors.

### Perceived clinical utility

The PBF anatomical model performed poorly across the board. All clinicians gave a rating of neutral or below for questions 1 to 5. Importantly, only 50% of the endocrinologists, 40% of the neurosurgeons and 0% of the ENT surgeons said they would like to use this anatomical model in clinical practice compared to 100% of all groups for the other types of anatomical models (see Fig. 8). Therefore, it seems unlikely that this type of anatomical model would be readily adopted into routine clinical practice. Specifically, the lack of colors depicting the different structures was considered a significant limitation, and is something which has been highlighted by other workers [23].

The MEX and MJ anatomical models were favoured by the endocrinologists while, in contrast, the VP anatomical model was preferred over the MEX and MJ anatomical models by the ENT surgeons. However, all three anatomical models were considered equivalent by the neurosurgical group. Importantly, although the MEX and MJ anatomical models were rated similarly by the endocrinologists, the MJ anatomical model cost almost five times as much as the MEX anatomical model, which is a potentially important consideration for clinical translation.

The ENT surgeons commented that the transparent bony material used in the VP anatomical model offered potential benefits as this allowed them to visualise the exact position of the carotid arteries through the bone - an important consideration when planning a transnasal surgical approach to the pituitary gland. The neurosurgeons commented in the questionnaire that the additional inclusion of the optic nerves and chiasm would be useful. The optic nerve is easily seen on the MR images and so could readily be included in future versions of these anatomical models. For example, the MEX printing method employed in this study is capable of using five different materials, whilst the MJ printing method offers even more color possibilities, and would therefore enable structures such as the optic nerves/chiasm to be printed in a discrete color. It is important to note however, in the case of the MEX printing method used here this would prohibit the use of dissolvable supports. A potential solution for this is to print some of the structures separately and retrospectively add them to the anatomical model [24]. We decided against this approach because of the risk of introducing errors in the relative positioning of the different structures when constructing the final anatomical model.

### Cost analysis

The cost of the printer is an important consideration when estimating the cost per model for the consumer printers and one which is potentially more complicated than can be fully captured in Table 1. For example, we have made two fundamental assumptions about each consumer printer (i) it needs to be purchased in full and (ii) it is solely used for printing these anatomical models. Both are not the case at our institution. However, if we take a conservative approach and consider the costs associated with creating 10 to 100 printed anatomical models, this provides a starting metric for comparison with the commercial printers. At 10 models per printer, the cost per model for VP and MEX would be £220 and £160 respectively and thereby highlighting that only a small number of models would need to be printed to make either of these options more financially viable than the commercial MJ printed anatomical model. Of course, it is also possible that the cost of the MJ prints would fall slightly in the context of a larger scale purchase of such models.

### Limitations

An important limitation of our work is that it was based on only a single representative clinical case. Nevertheless, this was a challenging and illustrative case, as the patient had undergone previous pituitary surgery and the suspected residual adenoma was at the lateral aspect of the pituitary fossa. In this work, our intention was to establish the feasibility, cost effectiveness and clinical acceptability of such 3D printed pituitary models and not to capture the full range of pre-surgical pituitary abnormalities, as reflected by 3D printed models. One of the next steps for this work will be to prospectively use anatomical models with patients to explore their usefulness in clinical practice as a tool for patient education and eliciting informed consent.

## Conclusion

In conclusion, we have shown that it is feasible to create accurate 3D anatomical models of pituitary tumors, together with the adjacent normal gland and surrounding critical structures (e.g. carotid arteries), using four different 3D printing techniques, each based on PET/CT coregistered with volumetric MRI. We have also demonstrated that clinicians from different specialties have overlapping, but discrete preferences for the different anatomical models based on specific priorities (e.g. ENT surgeons identified additional value from the VP anatomical model with its transparency). Importantly, the consumer printing techniques (VP and MEX) performed just as well as the commercial printing techniques (MJ and PBF), and were considerably less expensive, with the potential for up-scaling and therefore more widespread adoption into routine clinical practice.

## Data Availability

The datasets used and/or analysed during the current study are available from the corresponding author on reasonable request.

## Abbreviations

ACTH: Adrenocorticotropic hormone
CT: X-ray computed omography
ENT: Otolaryngology - ear, nose and throat
FSH: Follicle-stimulating hormone
FSPGR: Fast Spoiled Gradient Echo
GH: Growth hormone
HU: Hounsfield unit
LH: Luteinizing hormone
MEX: Material Extrusion
MJ: Material Jetting
MRI: Magnetic resonance imaging
PA: Pituitary adenoma
PBF: Powder Bed Fusion
PET: Positron Emission Tomography
STL: Standard Tessellation Language
TSH: Thyroid stimulating hormone
TSS: Transsphenoidal surgery
VP: Vat photopolymerization
